# Overexpression of c‐Jun inhibits erastin‐induced ferroptosis in Schwann cells and promotes repair of facial nerve function

**DOI:** 10.1111/jcmm.17241

**Published:** 2022-02-22

**Authors:** Dekun Gao, Yuyu Huang, Xiayu Sun, Jun Yang, Jianyong Chen, Jingchun He

**Affiliations:** ^1^ Department of Otorhinolaryngology‐Head and Neck Surgery Xinhua Hospital Shanghai Jiaotong University School of Medicine Shanghai China; ^2^ Shanghai Jiaotong University School of Medicine Ear Institute Shanghai China; ^3^ Shanghai Key Laboratory of Translational Medicine on Ear and Nose diseases Shanghai China

**Keywords:** c‐Jun, facial nerve injury, ferroptosis, NRF2/HO‐1 pathway, Schwann cells

## Abstract

Myelin undergoes various changes after nerve injury, and c‐Jun has a close relationship with Schwann cells (SCs). However, it remains unclear whether c‐Jun can be involved in nerve repair by regulating ferroptosis. To explore this, we first set up a facial nerve injury model and detected the changes of ferroptosis‐related proteins and c‐Jun by immunofluorescence and Western blot. Then, we cultured RSC 96 and pSCs, and studied the potential regulatory relationships by a combination of experimental methods such as CCK‐8, ELISA, immunofluorescence, qRT‐PCR, Western blot and viral transfection. Finally, we corroborated the role of c‐Jun through animal experiments. Our experiments revealed that ferroptosis occurs after facial nerve injury. Erastin decreased GPX4, c‐Jun proteins and GSH content, while PTGS2, NRF2, HO‐1 proteins, MDA, Fe^2+^ and ROS contents increased. This effect was inhibited after c‐Jun overexpression but was reversed after the addition of c‐Jun siRNA. Besides, we proved in vivo that c‐Jun could inhibit ferroptosis and promote the recovery of facial nerve function. In conclusion, our study identified the relationship between c‐Jun and ferroptosis during peripheral nerve injury repair, which provides new ideas for studying peripheral nerve injury and repair.

## 1 INTRODUCTION

Schwann cells (SCs), the myelin‐forming cells of the peripheral nerve, play an important role in the repair process of nerve injury.^1^ Currently, it is generally believed that SCs can reprogram to produce a cellular phenotype that promotes regeneration and repair after nerve injury, thus clearing excess myelin sheath, attracting macrophages, supporting the survival of damaged neurons, secreting various cytokines and guiding the growth of axons.^2^

However, the normal performance of these roles needs enough SCs, especially when the peripheral nerve is seriously damaged. The facial nerve is one of the key mixed nerves, which shares the motor and sensory functions. Clinically, facial nerve injury is not only difficult to treat but also causes serious psychological and social loads to patients and their families.^3^ Early studies suggested that nerve injury triggers the proliferation of SCs, but recent studies have differed from this view. Wagstaff found the number of SCs increased approximately 2.5‐fold after sciatic nerve injury and was essentially flat at 1 and 1.5 months but decreased by about 30% between 2 weeks and 2.5 months.^4^ Siironen found a threefold to fourfold increase at 1–2 weeks, with a sharp decline between 1.5 and 2 months.^5^ Salonen found the number of SCs after nerve injury was still 2–3 times higher than normal nerves at 2.5 months.^6^ While Jonsson found the number of SCs isolated from nerves was only 10–15% of that at 4 weeks after 6 months of chronic denervation.^7^ These studies collectively identified insufficient SCs as an important reason for the unsatisfactory restoration results. Therefore, inhibiting the death of SCs is a promising strategy for nervous diseases.

Ferroptosis is the latest cell death which differs from generally regulated cell death modes such as necrosis and apoptosis. It is characterized by iron‐dependent, lipid peroxidation and glutathione peroxidase depletion.^8^ When ferroptosis occurs, the nucleus is generally unchanged, intracellular mitochondrial volume decreases, and membrane density increases accompanied by intracellular ROS aggregation.

Many studies have reported the important role of ferroptosis in developing central nervous system diseases, such as stroke, Parkinson's disease, Alzheimer's disease and Huntington's disease.^9^, ^10^, ^11^, ^12^ Markers of ferroptosis, including increased lipid peroxidation, decreased GSH, reduced GPX4 activity and upregulation of PTGS2, are closely associated with neuronal cell death. Human differentiated neurons are sensitive to the occurrence of ferroptosis. Both ferrostatin‐1 and the iron chelator deferiprone reduced neuronal degeneration in animal models and improved function in these patients. Literature has reported that inhibiting the process of ferroptosis can improve the pain threshold of rats in the pathological pain model.^13^ Apart from this, there are still few studies on ferroptosis in peripheral nerve diseases.

C‐Jun is a key regulator of the response of SCs to peripheral nerve injury.^14^ The expression of c‐Jun is low or even absent in normal nerves, but rises rapidly after nerve injury, initiating a myelin repair program that includes increased trophic support for neurons, accelerated autophagy and breakdown of unwanted myelin, promoting Schwann cell elongation and formation of Bungner bands.^15^

The axonal regeneration and survival of neurons are severely impaired in the presence of c‐Jun inactivation. In addition, the expression of various neurotrophic factors was also significantly decreased.^16^ In contrast, c‐Jun overexpression in SCs significantly promotes axonal regeneration during the process of nerve injury repairing.^17^ Given that c‐Jun plays a vital role in peripheral nerve injury and repair, its role with ferroptosis remains unclear. It is necessary to study the relationship between c‐Jun and ferroptosis in SCs.

Here, we first investigated the changes expressions of c‐Jun and ferroptosis‐related proteins after facial nerve injury and then treated SCs with different concentrations of erastin to find a suitable concentration for subsequent experiments. After that, we further overexpressed and reduced c‐Jun expression to investigate changes in cellular ferroptosis‐related markers and the NRF2/HO‐1 pathway to demonstrate the role of c‐Jun in this process. Finally, we again validated the effect of c‐Jun on the ferroptosis of the facial nerve at the animal level. In a word, our observations provide a molecular basis for understanding the role of ferroptosis during facial nerve injury, and further support for the concept of glial repair of the peripheral nerve injury.

## 2 MATERIALS AND METHODS

### 2.1 Cell Culture, experimental animals and Reagents

RSC 96 were purchased from the Cell Bank of the Chinese Academy of Sciences. Primary Schwann cells (pSCs) were extracted from rats within 3 days of birth, as described in the previous publication.^18^ High‐glucose DMEM (Hyclone, USA) and DMEM/F‐12 (Hyclone, USA) medium containing10% foetal bovine serum (Gibco, USA) were used to culture RSC 96 and pSCs respectively. Cells were grown in a constant temperature incubator at 37 ℃ with 5% CO_2_. The animals used in this experiment were Sprague‐Dawley rats, weighing between 180 and 200g. The animals lived 12 h a day and 12 h a night, with sufficient water and food. All animal procedures were performed according to a protocol approved by the Institutional Animal Care and Use Committee of Shanghai Jiao Tong University School of Medicine.

The S‐100 antibody was purchased from Cell Signaling Technology (Beverly, MA, USA). CCK8, reactive oxygen species (ROS) kit, PI staining kit, RIPA, BCA kit and HRP‐labelled secondary antibodies were purchased from Beyotime (Shanghai, China). Hoechst 33342/PI cell staining kit was purchased from X‐Y Biotechnology (Shanghai, China). TRIzol reagents were purchased from Takara (Invitrogen, Carlsbad, CA, USA). C‐Jun, GPX4, PTGS2, NRF2 and HO‐1 antibodies were purchased from Abclonal (Wuhan, China), and β‐actin antibody was purchased from Beyotime (Shanghai, China). Erastin was purchased from Selleck Chemicals (Shanghai, China). Lentivirus, AAV and siRNA were provided by Hanheng Biological Co (Hangzhou, China).

### 2.2 Immunofluorescence

RSC 96 and pSCs were inoculated in 6‐well plates at a density of 20,000 cells/well and given different treatments after overnight adhesion. The cells were fixed at room temperature with 4% paraformaldehyde for 15 min, and the nerve tissue was fixed directly by soaking in paraformaldehyde. Cells can be directly incubated with antibodies after closure, but neural tissue requires additional embedding, sectioning, gradient alcohol dehydration and antigen repair before antibody incubation. Primary antibodies were added at 4℃ overnight, followed by the addition of secondary antibodies for 2 h at room temperature the next day. Then, DAPI was added to stain the nuclei for 5 min. After washing again, cell crawls and tissue sections were observed under a fluorescent microscope and photographed.

### 2.3 Cell viability experiment

RSC 96 and pSCs were inoculated in 96‐well plates at a density of 5000 cells/well. The next day the medium was replaced with a new one containing different concentrations of erastin (0, 0.2, 0.5, 1, 1.5, 2 and 2.5 μM). After 24 h, 10 μL CCK8 reagent was added and incubated at 37 degrees for 1 h. The absorbance at 490 nm was measured by a microplate reader. The survival rate of cells under the effect of different concentrations of erastin was calculated based on the measured OD values.

### 2.4 PI staining

After RSC 96 and pSCs have adhered in 6‐well plates, 1 μM erastin was added to the culture medium for 24 h. Then, the medium was discarded and 5ul of Hoechst 33342 and 5ul of PI staining solution were added directly. The cells were gently mixed and incubated for 30 min at 4°C. After staining, the cells were washed with PBS and then observed under a fluorescent microscope.

### 2.5 Reactive Oxygen Species (ROS) experiment

After treatment of RSC 96 and pSCs with 1 μM erastin, the cell culture medium was removed and DCFH‐DA diluted 1:1000 with serum‐free culture medium (final concentration of 10 µmol/L) was added to fully cover the cells and then incubated for 20 min in a 37°C incubator. The cells were washed three times with a serum‐free cell culture medium to fully remove the DCFH‐DA that had not entered the cells. Finally, the cells were observed under a fluorescence microscope.

### 2.6 Enzyme‐linked immunosorbent experiment

After RSC 96 and pSCs were cultured in 6‐well plates and treated accordingly, the supernatant was discarded and the cells were added to liquid nitrogen and frozen repeatedly for 3 times, and the dissociated solution was taken and the MDA, GSH and Fe^2+^ contents were determined by Enzyme‐linked immunosorbent experiment (ELISA) kit (MDA, GSH kits purchased by MEIMIAN, Jiangsu, China; Fe^2+^ kit purchased by Abcam, Cambridge, UK) according to the manufacturer's instructions.

### 2.7 Lentivirus transfection

For lentivirus construction, the c‐Jun‐gene cDNA cloned by PCR has been inserted into CMV‐MCS‐IRES‐puromycin lentiviral vectors. The recombinant lentivirus with c‐Jun‐gene coding sequence was produced by co‐transfection of 293T cells with plasmids PSPAX2 and PMD2G with LipoFiter TM. Lentivirus‐containing supernatant was harvested 48 h after transfection and filtered through 0.22‐μm cellulose acetate filters (Millipore, USA). Recombinant lentiviruses were concentrated by ultracentrifugation (2 h at 50,000 × *g*). To establish stable c‐Jun‐gene‐overexpressing cell lines, RSC 96 and pSCs were transduced with a lentiviral vector at an MOI of approximately 10 in the presence of 5 μg/ml polybrene. After 24 h, the culture medium was removed and a fresh medium was added to the RSC 96 and pSCs. 72 h after transduction, puromycin was added to the medium at the concentration of 5 mg/ml for stable cell line selection. The empty lentivector lenti‐puromycin was used as a negative control. After antibiotic selection for 3 weeks, stable overexpressing c‐Jun‐gene cells were obtained. After the cell was harvested, the expression level of c‐Jun‐gene was determined by Western blot and real‐time PCR.

### 2.8 Knockdown of c‐Jun

The small interfering RNAs (siRNAs) were transfected into RSC 96 and pSCs by polybrene following the manual instructions precisely. For gene silence, the sequences of c‐Jun siRNA are GAAAGCUGAUUACUGUCUAUA (siRNA‐1); GUGGCACAGCUUAAACAGAAA (siRNA‐2); and GCUACAGUAACCCUAAGAU (siRNA‐3). Cells transfected with negative control siRNA (UUCUCCGAACGUGUCACGU) were treated as a control.

### 2.9 Western Blot experiment

The treated cells were washed with PBS three times, then 200 μL RIPA lysate containing protease inhibitors PMSF and cocktail was added and incubated on ice for 15 min, and then, the lysate was collected and centrifuged at 12000 rpm for 15 min. The supernatant was collected and centrifuged for 15 min at 12000 rpm. The concentration of the supernatant was determined by the BCA kit, and 5X loading buffer was added to the remaining supernatant and boiled at 100 ℃ for 10 min to denature the protein. A protein of 20 μg was loaded on polyacrylamide gel for electrophoresis and then transferred to PVDF membranes (Thermo Fisher, USA). After blocking with 5% non‐fat milk in TBST, the membranes were incubated with anti‐c‐Jun, PTGS2, anti‐GPX4 and anti‐β‐actin at 4°C overnight, followed by incubating with HRP‐conjugated secondary antibody at room temperature for 1 hour, washed with TBST, added enhanced chemiluminescent substrates (Millipore, Billerica, MA) and luminescent on Image Lab System (Bio‐Rad, USA).

### 2.10 Reverse transcription‐polymerase chain reaction

Trizol was added to the cells and tissues washed with pre‐cold PBS, which were lysed on ice for 10 min, and then, 1/5 volume of chloroform was added. After blending, the mixture was centrifuged at 4℃ for 15 min at 12000 rpm. The supernatant was added to an equal volume of isopropanol and centrifuged at 4 ℃ for 15 min again. Wash the precipitate twice with 75% alcohol and finally dissolve the precipitate with DEPC water. The reverse transcription kit was used to synthesize cDNA. Reverse transcription‐polymerase chain reaction (RT‐PCR) on‐machine operation (ABI7500) was performed according to the instructions of the SYBR Green quantitative PCR kit. The primers were as follows:

c‐Jun Forward GCTGAGTGTCTGTATGCTGGG.

c‐Jun Reverse GGCGTGCTTGAGCAGAAGTT.

β‐actin Forward CCCATCTATGAGGGTTACGC.

β‐actin Reverse TTTAATGTCACGCACGATTTC.

Reaction conditions were as follows: 5 min at 95°C, followed by 40 cycles of 30 s. at 95°C, 30 s. at 57°C and 30 s. at 72°C. The expression level of the target gene was calculated by 2^−ΔΔCT^.

### 2.11 Facial nerve injury model and AAV injection

The rat model was established, and the facial nerve function was evaluated based on published article.^18^ Briefly, rats were anaesthetized by intraperitoneal injection of 1% pentobarbital sodium at a dose of 40 mg/kg. After successful anaesthesia, the right posterior auricular incision was made, and the main facial nerve and its three branches were dissected and exposed, and we then used a microinjector to inject the AAV into the neural tissue at this time and tested the effect of virus expression after 3 weeks. As for the nerve injury model, the facial nerve trunk was clamped with a vascular clamp for 50 seconds.

### 2.12 Facial nerve scoring

The rat model was established, and the facial nerve function was evaluated based on the published article.^18^ The facial nerve function contains two aspects: Vibrissae observation, eye closing and blinking reflex observation. The specific scoring criteria are as follows: The absence of eye blinking and closure scored 1; the presence of orbicular muscle contraction without blinking reflex scored 2; 50% of eye closure through blinking reflex scored 3; 75% of closure scored 4. The presence of complete eye closure and blinking reflex scored 5. The absence of movement and posterior position of the vibrissae scored 1; slight shivering and posterior position scored 2; greater shivering and posterior position scored 3; normal movement with a posterior position scored 4; and the symmetrical movement of the vibrissae, with an anterior position, scored 5.

### 2.13 Statistical analysis

Data were presented as mean ± standard deviation (SD). Graphpad Prism 8.0 software is used for statistical analysis of the obtained data. All data were obtained by at least three independent experiments. Two‐tailed, unpaired Student's t‐tests were used to determine statistical significance when comparing two groups. One‐way ANOVA followed by a Dunnett multiple comparisons test was used when comparing more than two groups. *p* < 0.05 was considered statistically significant.

## 3 RESULTS

### 3.1 Ferroptosis occurs after facial nerve injury and dynamic changes in c‐Jun expression

**FIGURE 1:**
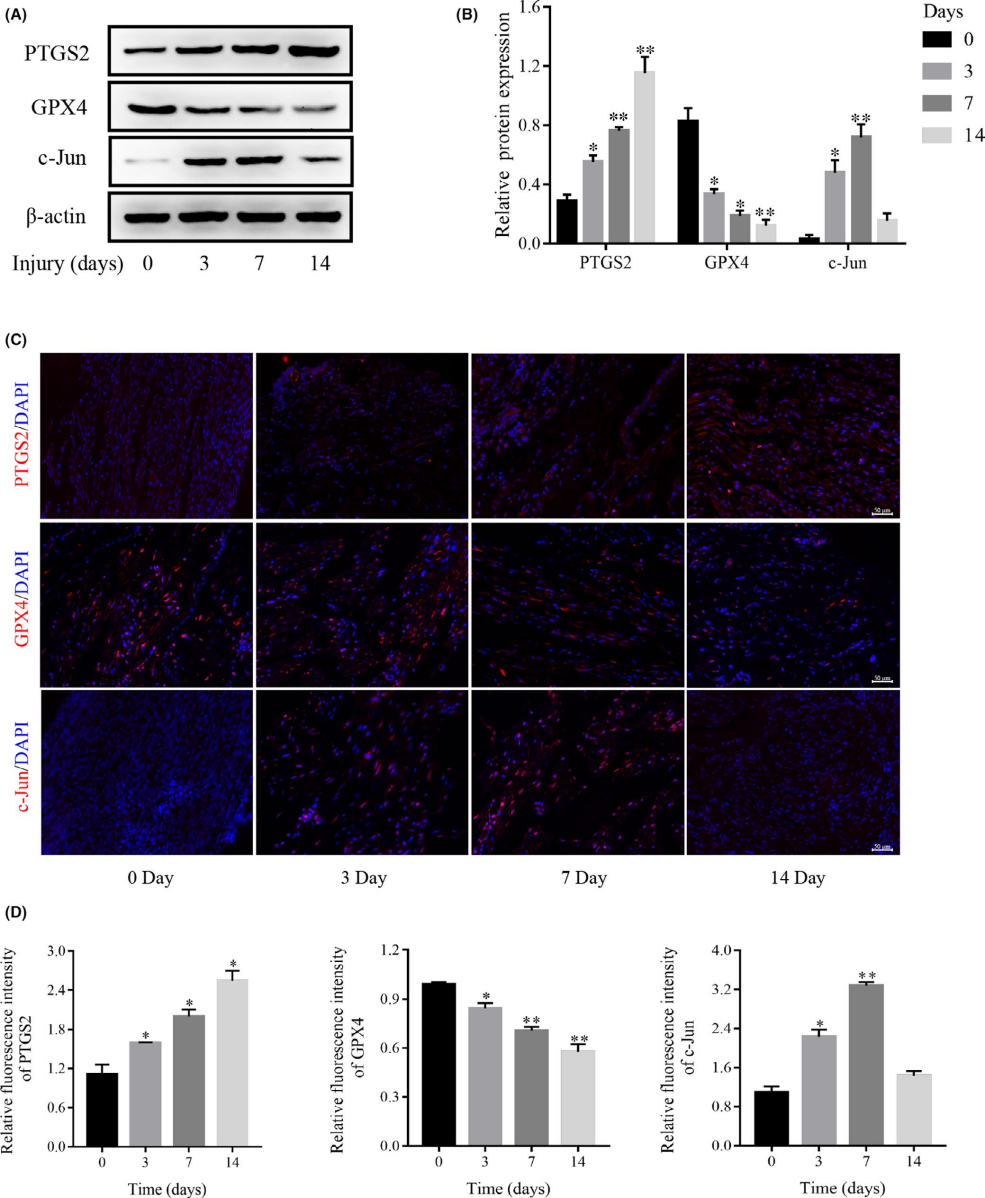
Ferroptosis occurs after facial nerve injury. (A) The expressions of GPX4, PTGS2 and c‐Jun proteins after facial nerve injury by Western blot. (B) Densitometric analysis of GPX4, PTGS2 and c‐Jun proteins relative expression. (C) The expression of GPX4, PTGS2 and c‐Jun after facial nerve injury by immunofluorescence. (D) Fluorescence intensity analysis of GPX4, PTGS2 and c‐Jun proteins relative expression. Data are presented as mean ± SD, and one‐way ANOVA was performed for statistical differences. All experiments were performed at least 3 times independently, *p < 0.05, **p < 0.01, compared to the Day 0

The changes of ferroptosis after facial nerve injury are unclear. To investigate whether ferroptosis occurs after facial nerve injury and its relationship with c‐Jun, Western blot and immunofluorescence experiments were used to detect changes in PTGS2, GPX4 and c‐Jun expressions at Days 3, 7 and 14 after facial nerve injury. We found that PTGS2 protein kept increasing and GPX4 protein kept decreasing after facial nerve injury, while c‐Jun protein started decreasing at 14 days after the first 7 days of increase (Figure 1A‐D). These results suggest that ferroptosis does occur, and c‐Jun may play an important role in the process of facial nerve injury and repair.

### 3.2 Erastin increases the death of RSC 96 and pSCs and the accumulation of intracellular ROS level

**FIGURE 2:**
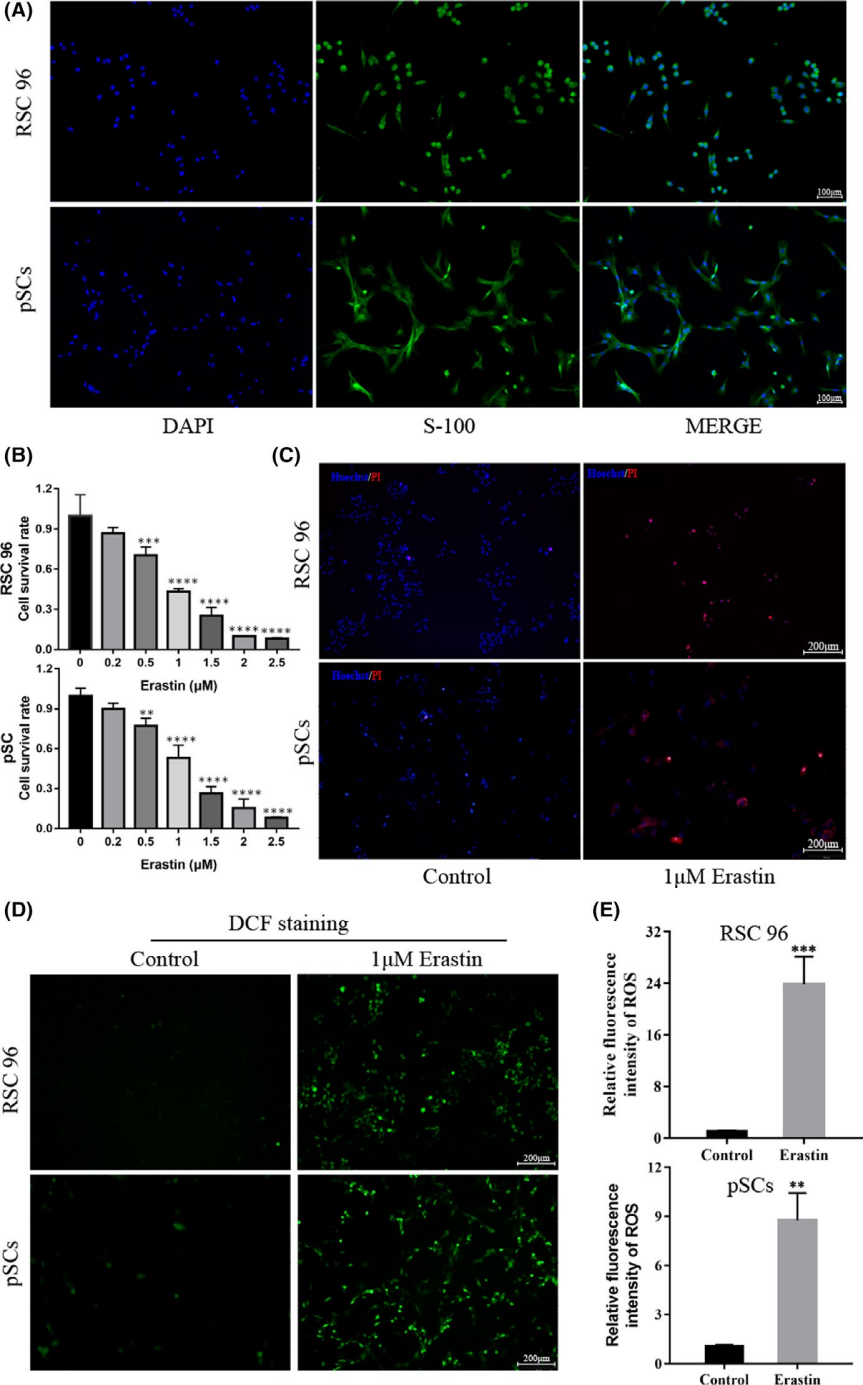
Erastin increases the death of Schwann cells and the accumulation of ROS. (A) The expression of the molecular marker S‐100 in RSC 96 and pSCs (scale bar =100 μm). (B) The viability of RSC 96 and pSCs decreased after incubation with erastin for 24 h by CCK‐8 assay. (C) Cell death was confirmed by PI and Hoechst 33342 staining for 24 h (scale bar =200 μm). (D) The effect of erastin on intracellular ROS accumulation in RSC 96 and pSCs by immunofluorescence (Scale bar =200 μm). (E) Fluorescence intensity analysis of intracellular ROS accumulation in RSC 96 and pSCs. Data are presented as mean ± SD. T‐test and one‐way ANOVA were performed for statistical differences. All experiments were performed at least 3 times independently, **p < 0.01, ***p < 0.001, ****p < 0.0001 compared to the control group

We first extracted pSCs from rats at 3 days of neonatal and identified the RSC 96 and pSCs as SCs with a purity of >99% by immunofluorescence (Figure 2A). Then, we investigated the effect of erastin on the viability of RSC 96 and pSCs. We cultured RSC 96 and pSCs in a medium containing different concentrations of erastin (0, 0.2, 0.5, 1, 1.5, 2 and 2.5 μM) for 24 h and found that erastin concentration‐dependently reduced the viability of RSC 96 and pSCs, where 1uM erastin reduced the viability of cells to approximately half of normal (Figure 2B). PI staining was also performed to confirm the role of 1 uM erastin in RSC 96 and pSCs (Figure 2C). In addition, the ROS content was detected by the ROS kit and we found ROS level was extremely low but increased significantly after adding erastin for 24 h (Figure 2D, E).

### 3.3 Erastin induces the ferroptosis of RSC 96 and pSCs

**FIGURE 3:**
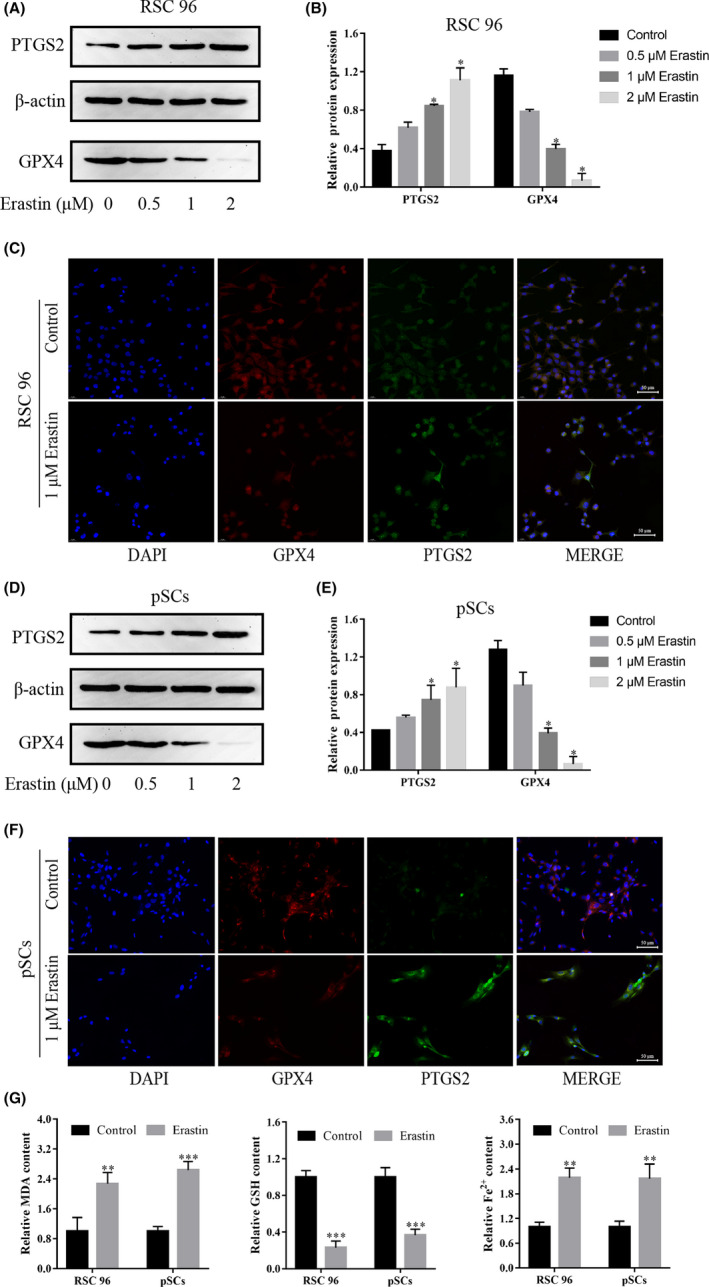
Erastin induces ferroptosis in RSC 96 and pSCs. (A, D) The expressions of GPX4 and PTGS2 proteins were induced by 0–2 μM erastin in RSC 96 and pSCs by Western blot. (B, E) Densitometric analysis of GPX4 and PTGS2 proteins relative expression in RSC 96 and pSCs. (C, F) The expression of GPX4 and PTGS2 induced by 1 μM erastin in RSC 96 and pSCs by immunofluorescence (scale bar =50 μm). (G) 1 μM Erastin decreased the GSH level and increased the MDA and Fe2+ level in RSC 96 and pSCs by ELISA. Data are presented as mean ±SD, and one‐way ANOVA and t‐test were performed for statistical differences. All experiments were performed at least 3 times independently, **p < 0.01, ***p < 0.001, ****p < 0.0001 compared to the control group

To further clarify the relationship between the death caused by erastin and ferroptosis, Western blot and immunofluorescence experiments were used to detect changes in the expressions of GPX4 and PTGS2 proteins. In addition, the ELISA experiment was also used to detect intracellular contents of MDA, GSH and Fe^2+^. The experimental results revealed that erastin dose‐dependently reduced the expression of GPX4 protein while increasing the expression of PTGS2 protein (Figure 3A‐F). In addition to this, erastin significantly increased the content of MDA and Fe^2+^ while decreasing the content of GSH (Figure 3G). Together, these results demonstrated that RSC 96 and pSCs undergo ferroptosis in response to erastin.

### 3.4 The NRF2/HO‐1 pathway and c‐Jun are involved in the ferroptosis of RSC 96 and pSCs

**FIGURE 4:**
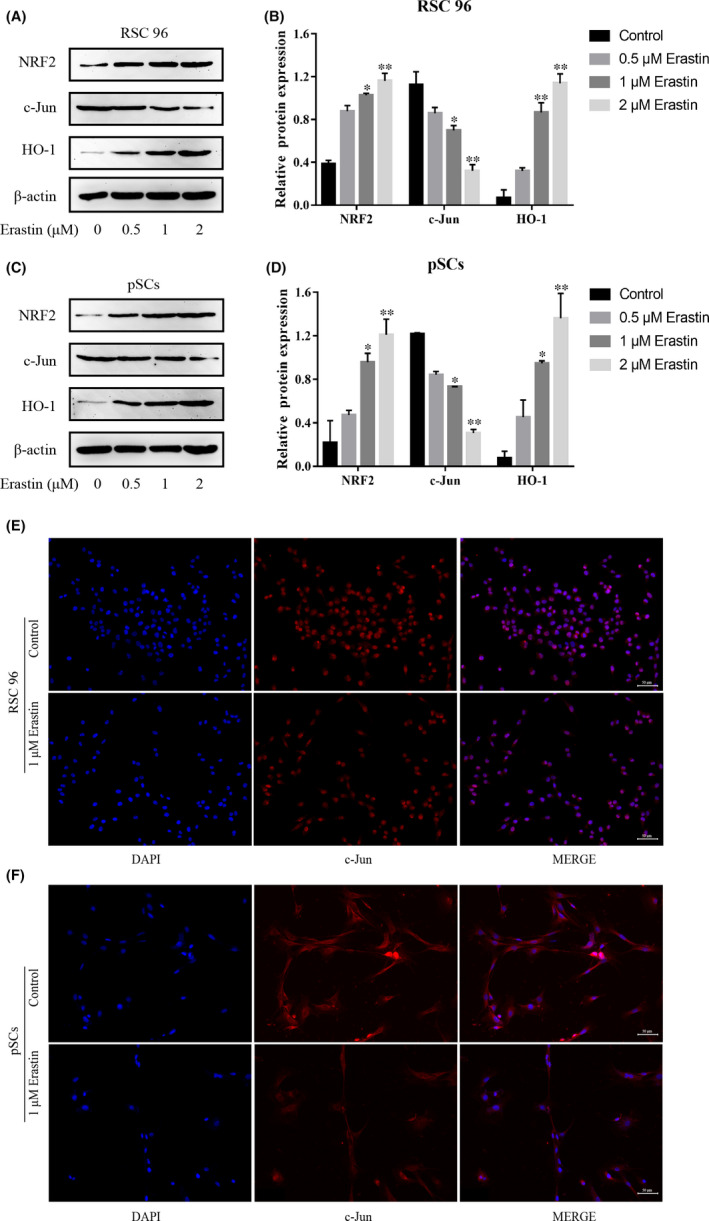
NRF2/HO‐1 pathway is involved in erastin‐induced ferroptosis in RSC 96 and pSCs. (A, C) The expression of NRF2/HO‐1, and c‐Jun protein in RSC 96 and pSCs by Western blot. (B, D) Densitometric analysis of NRF2/HO‐1 and c‐Jun proteins relative expression in RSC 96 and pSCs. (E, F) Immunofluorescence showed that the expression of c‐Jun in RSC 96 and pSCs induced by 1 μM erastin (scale bar =50 μm). Data are presented as mean ± SD, and one‐way ANOVA was performed for statistical differences. All experiments were performed at least 3 times independently, *p < 0.05, **p < 0.01, compared to the control group

NRF2/HO‐1 pathway is a classical pathway during the occurrence of ferroptosis. We detected the expressions of NRF2, HO‐1 and c‐Jun proteins in erastin‐treated RSC 96 and pSCs by Western blot and immunofluorescence experiments. The experimental results revealed that erastin dose‐dependently reduced the expression of the c‐Jun protein while increasing the expressions of NRF2 and HO‐1 proteins (Figure 4A‐F), which means that NRF2/HO‐1 pathway and c‐Jun is involved in the ferroptosis.

### 3.5 Overexpression of c‐Jun reduces erastin‐induced ferroptosis in RSC 96 and pSCs

**FIGURE 5:**
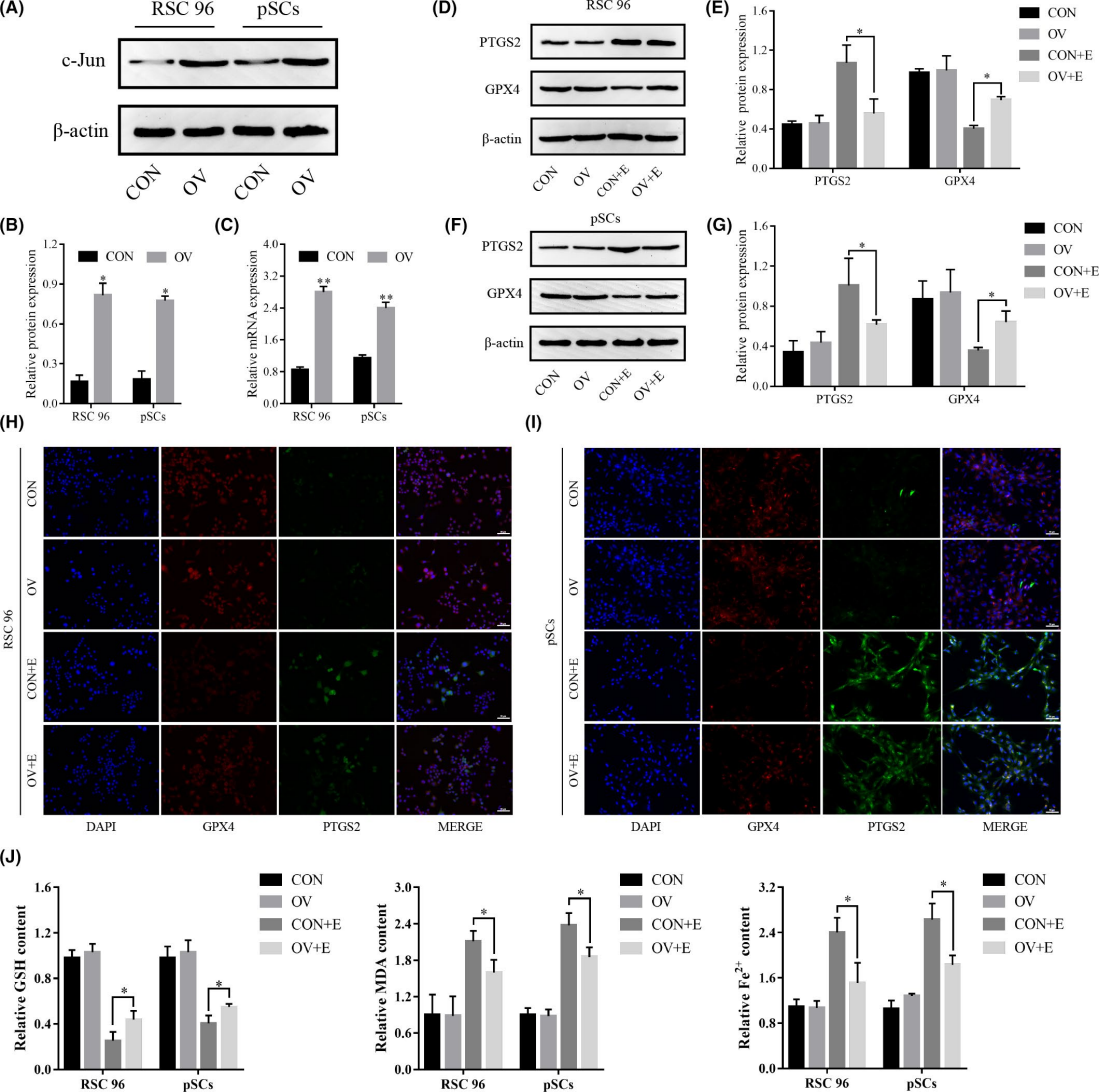
Overexpression of c‐Jun inhibits erastin‐induced ferroptosis in RSC 96 and pSCs. (A) The expression of c‐Jun protein after lentivirus transfection by Western blot. (B) Densitometric analysis of c‐Jun protein. (C) The expression of c‐Jun mRNA after lentivirus transfection by real‐time quantitative PCR. (D, F) The expression of GPX4 and PTGS2 proteins after overexpression of c‐Jun in RSC 96 and pSCs by Western blot. (E, G) Densitometric analysis of GPX4 and PTGS2 proteins. (H, I) The expression of GPX4 and PTGS2 proteins after overexpression of c‐Jun in RSC 96 and pSCs by immunofluorescence. (J) The expression level of GSH, MDA and Fe2+ after overexpression of c‐Jun by ELISA. Data are presented as mean ± SD, and one‐way ANOVA and t‐test were performed for statistical differences. All experiments were performed at least 3 times independently, *p < 0.05, compared to the control group

The expression of c‐Jun protein was decreased when erastin induced ferroptosis in RSC 96 and pSCs, but the regulatory relationship between ferroptosis and c‐Jun remains unclear. To clarify this issue, we overexpressed c‐Jun in RSC 96 and pSCs by lentivirus. Western blot and RT‐qPCR results demonstrated that we successfully overexpressed c‐Jun protein in RSC 96 and pSCs (Figure 5A, B and C). After that, we added 1 uM erastin to c‐Jun overexpressing cells (OV) and control cells (CON) in RSC 96 and pSCs, respectively, to detect changes in the expression of ferroptosis‐related proteins and intracellular factors. The results showed that CON and OV group cells expressed similar protein levels, but GPX4 protein expression decreased and PTGS2 protein expression increased after erastin treatment, and the changes were significantly less in the OV+E group than in the CON+E group (Figure 5D‐G). Followed with immunofluorescence, the results were the similarity to the Western blot. The fluorescence intensity of GPX4 and PTGS2 protein was almost the same in the CON group and OV group, but the fluorescence intensity of GPX4 protein was significantly decreased and PTGS2 protein was significantly increased in the CON+E and OV+E groups. The changing trend of immunofluorescence intensity in the OV+E group was less than that in the CON+E group (Figure 5H, 5I). In addition, the c‐Jun OV group expressed higher GSH content and lower MDA and Fe^2+^ contents compared with the CON group (Figure 5D‐5J), which confirmed the protective effect of c‐Jun on cellular ferroptosis in RSC 96 and pSCs.

### 3.6 C‐Jun inhibits ferroptosis through the NRF2/HO‐1 pathway

**FIGURE 6:**
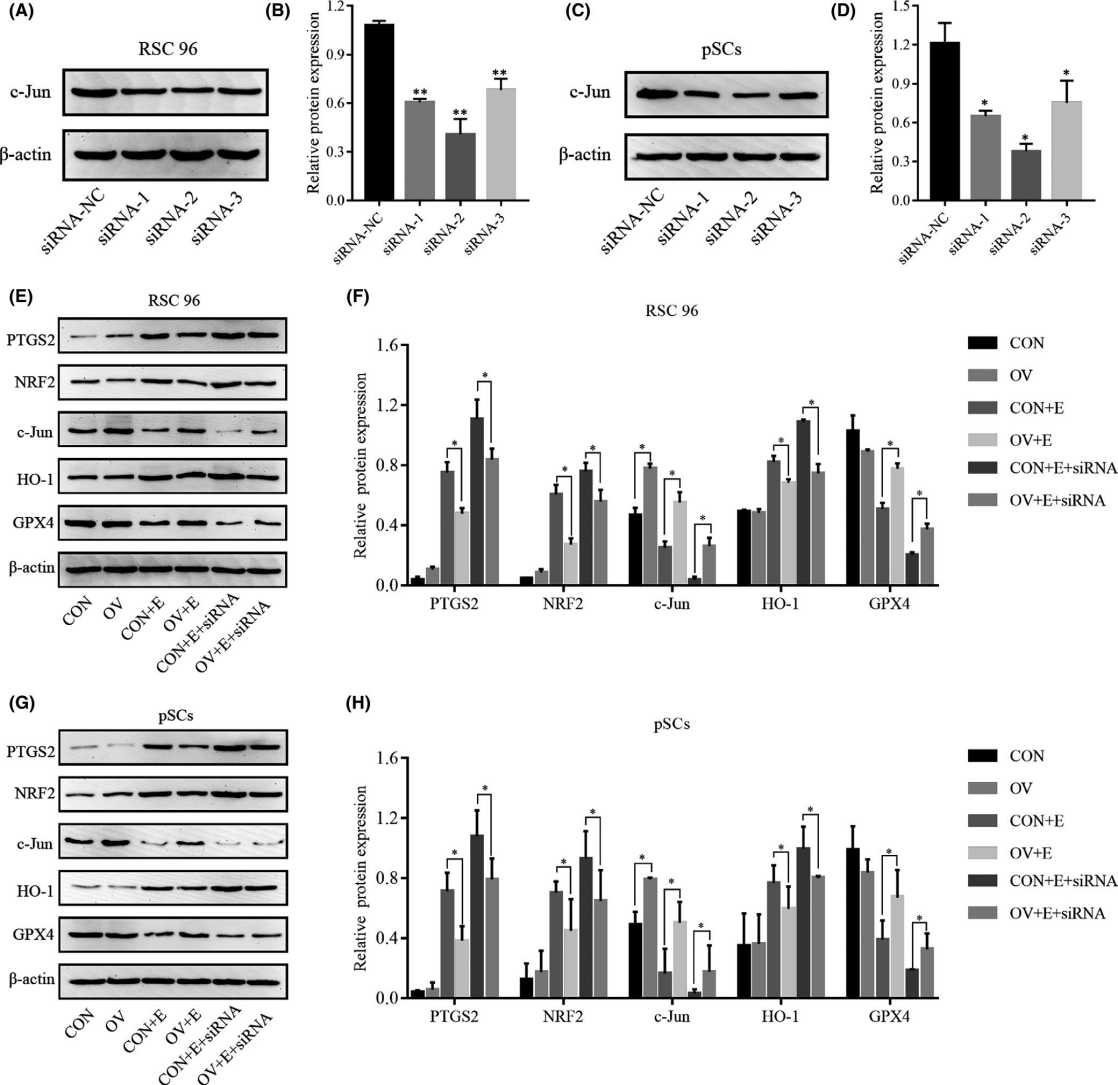
C‐Jun inhibits erastin‐induced ferroptosis via the NRF2/HO‐1 pathway. (A, C) The expression of the c‐Jun protein in RSC 96 and pSCs after siRNA transfection by Western blot. (B, D) Densitometric analysis of c‐Jun protein. (E, G) The expression of PTGS2, NRF2, c‐Jun, HO‐1 and GPX4 proteins with or without siRNA in c‐Jun overexpression and control RSC 96 and pSCs. (F, H) Densitometric analysis of G PTGS2, NRF2, c‐Jun, HO‐1 and GPX4 proteins. Data are presented as mean ± SD, and one‐way ANOVA was performed for statistical differences between groups. All experiments were performed at least 3 times independently, *p < 0.05, **p < 0.01, compared to the control group

To investigate the relationship among c‐Jun, NRF2/HO‐1 pathway, and ferroptosis in RSC 96 and pSCs. We used siRNA to reduce the expression of c‐Jun to see whether it could rescue the overexpression effect of c‐Jun and its effect on the expressions of NRF2, HO‐1, PTGS2 and GPX4 proteins. Western blot experiments demonstrated that siRNA‐1, siRNA‐2 and siRNA‐3 all reduced the expression of the c‐Jun protein in RSC 96 and pSCs, with siRNA‐2 having a more significant effect (Figure 6A‐D), so we used siRNA‐2 in our subsequent experiments. After adding siRNA to c‐Jun OV cells, we found that the protective effect of c‐Jun against ferroptosis in RSC 96 and pSCs was reduced and the expressions of NRF2, HO‐1 proteins were increased, suggesting that c‐Jun could inhibit ferroptosis by suppressing the NRF2/HO‐1 pathway (Figure 6E‐H).

### 3.7 C‐Jun inhibits the ferroptosis of the facial nerve and promotes the repair of facial nerve function

**FIGURE 7:**
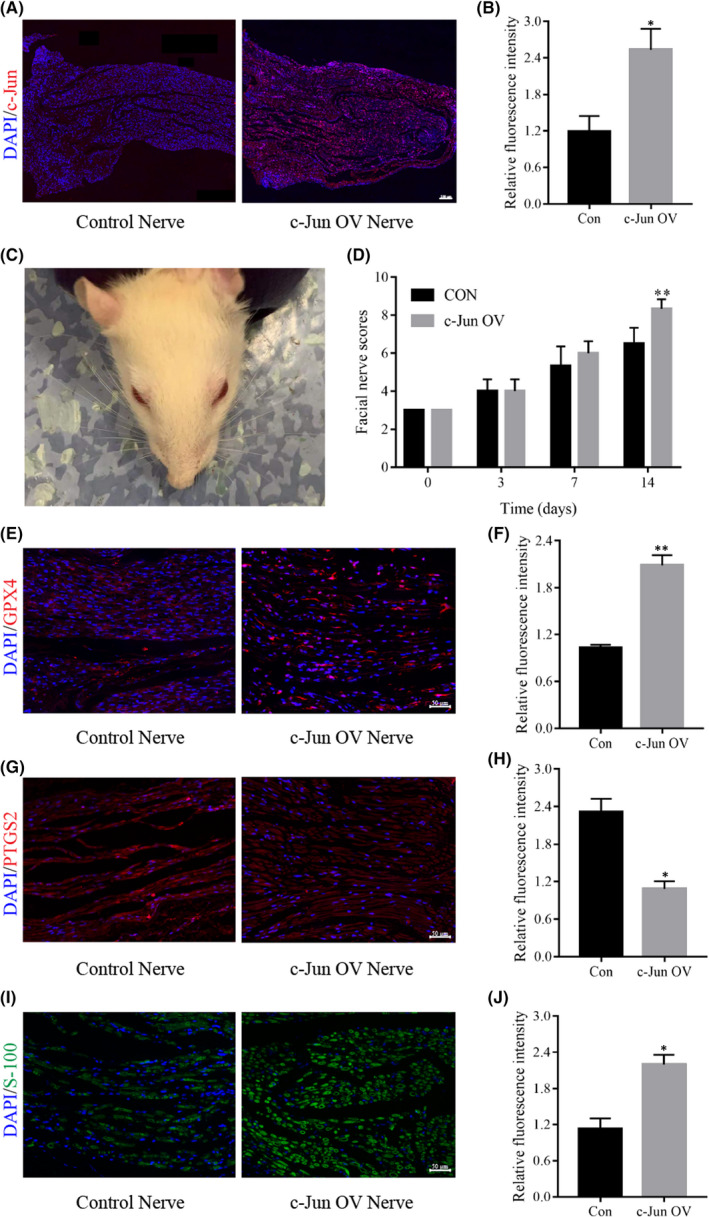
Overexpression of c‐Jun improves facial nerve function and inhibits the ferroptosis of the facial nerve. (A) The successful construction of a facial nerve c‐Jun overexpression model by immunofluorescence. (B) Fluorescence intensity analysis of c‐Jun protein relative expression. (C) A typical model of facial nerve injury in rats. (D) Scoring at different time points after facial nerve injury. (E, G, and I) The expression of GPX4, PTGS2 and S‐100 proteins after facial nerve at 14 days by immunofluorescence. (F, H and J) Fluorescence intensity analysis of GPX4, PTGS2 and S‐100 proteins. Data are presented as mean ± SD. T‐test and one‐way ANOVA were performed for statistical differences between groups. All experiments were performed at least 3 times independently, *p < 0.05, **p < 0.01, compared to the control group

At the animal level, we overexpressed c‐Jun in the facial nerve to observe its effect on ferroptosis and the recovery of facial nerve function. By injecting an adeno‐associated virus into the facial nerve, immunofluorescence experiments after 3 weeks revealed that the facial nerve in the experimental group expressed abundant c‐Jun protein, while the facial nerve in the control group expressed almost none (Figure 7A, 7B). After determining that c‐Jun was successfully overexpressed, we constructed a model of facial nerve injury (Figure 7C) and scored the function of the facial nerve at 3, 7 and 14 days after injury respectively. The scoring results revealed that the facial nerve scores in the c‐Jun overexpression group were better than those in the control group after 7 days, and the scores at 14 days were statistically significant (Figure 7D). Immunofluorescence experiments of facial nerve tissues from the experimental and control groups were performed after 14 days of facial nerve scoring, and we found that GPX‐4 and S‐100 proteins were significantly increased after c‐Jun overexpression (Figure 7E, F, I and J), while PTGS2 protein expression was significantly decreased (Figure 7G, H). These results suggested that overexpression of c‐Jun can inhibit nerve ferroptosis and promote the repair of facial nerve function.

## 4 DISCUSSION

Facial nerve injury can lead to various complications, including dry eyes, keratitis, speech and pronunciation disorders, eating difficulties, and facial expression disorders, severely affecting the patient's mental health and social activities.^19^ The role of SCs in nerve injury repair has been gradually recognized in recent years, and their proliferation, migration, diversification and death are closely related to the repairing of injured nerves.^1^

Previous studies found, c‐Jun and p75NTR, two major genes associated with the repair of SCs in rodents, were also upregulated in acutely injured human nerves, while the expression of these two genes decreases during long‐term denervation.^20^ In rodents, reduced levels of c‐Jun and p75NTR marks stalled nerve repairing, which is also considered a major obstacle to effective nerve repair. Upregulation of c‐Jun is a central factor in the reprogramming process that occurs in SCs after nerve injury and is essential for successful nerve regeneration.^14^, ^15^, ^16^ However, the upregulation of c‐Jun does not persist during acute injury, lasting only 7–10 days.^21^ Consistent with previous studies, our study found that c‐Jun increased consistently at 7 days after facial nerve injury, with a decrease at 14 days. We also found that c‐Jun was downregulated in RSC 96 and pSCs after the addition of erastin, a small fractional compound that triggers ferroptosis. The inconsistent changes in c‐Jun protein expression in animal and cellular experiments may be related to the fact that animal experiments are faced with more complex regulation. One of the key factors is the neuron. After peripheral nerve injury, the function of neurons shifts mainly from cell–cell communication to activation of related genes regulating the function of SCs to promote axonal regeneration.^22^, ^23^ In addition to this, there are invasive macrophages and various inflammatory factors that may affect axonal repair and regeneration.^24^ A combination of factors may lead to such differences, and thus, c‐Jun gradually decreases after a short period of high expression.

In normal conditions, the reverse glutamate/cystine transporter (System Xc‐) is responsible for the intracellular transport of glutamate and extracellular transport of cystine.^25^ The cystine transported into the cell is used for the synthesis of intracellular GSH, which acts as a co‐regulator of GPX4, helping convert reduced glutathione disulfide and lipid hydroperoxides or hydrogen peroxide to alcohol or water to scavenge lipid peroxides.^26^ Erastin is a ferroptosis inducer that promotes ferroptosis by inhibiting the action of System Xc‐. PTGS2, a key enzyme in prostaglandin biosynthesis, plays the role of both dioxygenase and peroxidase and serves as one of the markers of ferroptosis.^27^ Both animal experiments and erastin‐induced cellular experiments showed a decrease in GPX4 protein expression and an increase in PTGS2 protein expression. This implies that we can use cellular experiments to model the changes in SCs after nerve injury. During erastin‐induced changes in RSC 96 and pSCs, we found a decrease in cell viability, along with a decrease in GSH content and an increase in ROS, MDA and Fe^2+^ contents. These results confirm the occurrence of cellular ferroptosis. At the same time, we also found that the NRF2/HO‐1 pathway was activated. NRF2/HO‐1 pathway is considered the key regulatory pathway of ferroptosis. Normally, NRF2 binds to Keap1 and continues to be inactivated by ubiquitination and degradation in the proteasome.^28^ Once the body is in oxidative stress, NRF2 is released from the Keap1 binding site and rapidly translocated to the nucleus, subsequently activating transcriptional pathways to balance oxidative stress and maintain cellular redox homeostasis.^29^

After overexpression of c‐Jun in RSC 96 and pSCs, we found no changes in the expressions of GPX4, PTGS2 proteins, and GSH, MDA and Fe^2^ ^+^ contents in both control and c‐Jun OV groups. A possible explanation is that exogenous introduction of c‐Jun genes for overexpression does not change the external environment to which the cells are exposed, and thus, these expressions are unchanged. In contrast, after the addition of erastin, c‐Jun OV group showed a significant inhibitory effect on ferroptosis.

Erastin, an exogenous compound, alters the external environment in which cells are exposed, making them difficult to defend against and thus gradually dying under normal circumstances, but the cells can resist this damage in the case of c‐Jun overexpression.

Furthermore, the protective effect of c‐Jun against erastin was manifested by added siRNA to the c‐Jun overexpressed RSC and pSCs. The results showed that both ferroptosis‐related proteins and NRF2/HO‐1 pathway proteins were partially reversed. Our study revealed that the NRF2/HO‐1 pathway was inhibited by c‐Jun overexpression, and thus exerts an anti‐ferroptosis effect. Many studies have found that NRF2 activation confers resistance to ferroptosis in cancer cells.^30^, ^31^, ^32^ Adedoyin found that HO‐1 inhibited erastin‐induced ferroptosis in proximal tubular epithelial cells in acute kidney injury.^33^ Kwon found that HO‐1 inhibitor reduced erastin‐induced ferroptosis in fibrosarcoma.^34^ These seemingly contradictory results imply a complex relationship between ferroptosis and the NRF2/HO‐1 pathway. The NRF2/HO‐1 pathway activated under different conditions has different roles, and the exact relationship needs to be further investigated.

In the final animal study, we observed that c‐Jun can inhibit the ferroptosis of the facial nerve, promote facial nerve function recovery and promote S‐100 expression, allowing the nerve to form a more morphological myelin sheath. This provides a new direction to explain the neuroprotective role played by c‐Jun, and we can intervene in neural damage and repair through the pathway of ferroptosis.

## 5 CONCLUSION

We found that ferroptosis occurs after facial nerve injury. c‐Jun, as a key nerve injury repair factor, can inhibit ferroptosis through the NRF2/HO‐1 pathway, which further refines the mechanism by which c‐Jun promotes nerve repair and provides a new direction for the repair of facial nerve injury.

## CONFLICT OF INTEREST

The authors declare that there are no known competing financial interests or personal relationships that could have appeared to influence this work.

## AUTHOR CONTRIBUTIONS

**Dekun Gao:** Conceptualization (lead); Formal analysis (lead); Methodology (lead); Software (lead); Validation (lead); Writing – original draft (lead). **Yuyu Huang:** Formal analysis (equal); Investigation (equal); Project administration (equal); Resources (equal); Supervision (equal). **Xiayu Sun:** Formal analysis (equal); Resources (equal); Supervision (equal). **Jun Yang:** Conceptualization (equal); Project administration (equal); Validation (equal); Writing – review & editing (equal). **Jianyong Chen:** Investigation (equal); Supervision (equal); Validation (equal). **Jingchun He:** Conceptualization (equal); Resources (lead).

## Data Availability

The data that support the findings are available from the corresponding author upon reasonable request.

## References

[1] Nocera G , Jacob C . Mechanisms of Schwann cell plasticity involved in peripheral nerve repair after injury. Cell Mol Life Sci. 2020;77(20):3977‐3989. 10.1007/s00018-020-03516-9 32277262 PMC7532964

[2] Jessen KR , Arthur‐Farraj P . Repair Schwann cell update: Adaptive reprogramming, EMT, and stemness in regenerating nerves. Glia. 2019;67(3):421‐437. 10.1002/glia.23532 30632639

[3] Condie D , Tolkachjov SN . Facial nerve injury and repair: a practical review for cutaneous surgery. Dermatol Surg. 2019;45(3):340‐357. 10.1097/DSS.0000000000001773 30640780

[4] Wagstaff LJ , Gomez‐Sanchez JA , Mirsky R , Jessen KR . The relationship between Schwann cell c‐Jun and regeneration failures due to ageing and long‐term injury. Glia. 2017;65:E532.

[5] Siironen J , Collan Y , Röyttä M . Axonal reinnervation does not influence Schwann cell proliferation after rat sciatic nerve transection. Brain Res. 1994;654(2):303‐311. 10.1016/0006-8993(94)90492-8 7987679

[6] Salonen V , Aho H , Röyttä M , Peltonen J . Quantitation of Schwann cells and endoneurial fibroblast‐like cells after experimental nerve trauma. Acta Neuropathol. 1988;75(4):331‐336. 10.1007/BF00687785 3364158

[7] Jonsson S , Wiberg R , McGrath AM , et al. Effect of delayed peripheral nerve repair on nerve regeneration, Schwann cell function and target muscle recovery. PLoS One. 2013;8(2):e56484. 10.1371/journal.pone.0056484 23409189 PMC3567071

[8] Dixon SJ , Lemberg KM , Lamprecht MR , et al. Ferroptosis: an iron‐dependent form of nonapoptotic cell death. Cell. 2012;149(5):1060‐1072. 10.1016/j.cell.2012.03.042 22632970 PMC3367386

[9] Liu J , Guo ZN , Yan XL , et al. Crosstalk Between Autophagy and Ferroptosis and Its Putative Role in Ischemic Stroke. Front Cell Neurosci. 2020;14(1):1‐15. 10.3389/fncel.2020.577403 33132849 PMC7566169

[10] Mahoney‐Sánchez L , Bouchaoui H , Ayton S , Devos D , Duce JA , Devedjian JC . Ferroptosis and its potential role in the physiopathology of Parkinson's Disease. Prog Neurogibol. 2021;196:101890. 10.1016/j.pneurobio.2020.101890 32726602

[11] Yan N , Zhang J . Iron metabolism, ferroptosis, and the links with Alzheimer's disease. Front Neurosci. 2019;13:1443. 10.3389/fnins.2019.01443 32063824 PMC7000453

[12] Mi Y , Gao X , Xu H , Cui Y , Zhang Y , Gou X . The emerging roles of ferroptosis in Huntington's disease. Neuromolecular Med. 2019;21(2):110‐119. 10.1007/s12017-018-8518-6 30600476

[13] Guo Y , Du J , Xiao C , et al. Inhibition of ferroptosis‐like cell death attenuates neuropathic pain reactions induced by peripheral nerve injury in rats. Eur J Pain. 2021;25(6):1227‐1240. 10.1002/ejp.1737 33497529

[14] Arthur‐Farraj PJ , Latouche M , Wilton DK , et al. c‐Jun reprograms Schwann cells of injured nerves to generate a repair cell essential for regeneration. Neuron. 2012;75(4):633‐647. 10.1016/j.neuron.2012.06.021 22920255 PMC3657176

[15] Jessen KR , Mirsky R . The repair Schwann cell and its function in regenerating nerves. J Physiol. 2016;594(13):3521‐3531. 10.1113/JP270874 26864683 PMC4929314

[16] Fontana X , Hristova M , Da Costa C , et al. c‐Jun in Schwann cells promotes axonal regeneration and motoneuron survival via paracrine signaling. J Cell Biol. 2012;198(1):127‐141. 10.1083/jcb.201205025 22753894 PMC3392945

[17] Huang L , Quan X , Liu Z , et al. c‐Jun gene‐modified Schwann cells: upregulating multiple neurotrophic factors and promoting neurite outgrowth. Tissue Eng Part A. 2015;21(7–8):1409‐1421. 10.1089/ten.tea.2014.0416 25588149 PMC4394874

[18] Gao D , Tang T , Zhu J , Tang Y , Sun H , Li S . CXCL12 has therapeutic value in facial nerve injury and promotes Schwann cells autophagy and migration via PI3K‐AKT‐mTOR signal pathway. Int J Biol Macromol. 2019;124:460‐468. 10.1016/j.ijbiomac.2018.10.212 30391592

[19] Bruins TE , van Veen MM , Mooibroek‐Leeuwerke T , Werker PMN , Broekstra DC , Dijkstra PU . Association of socioeconomic, personality, and mental health factors with health‐related quality of life in patients with facial palsy. JAMA Otolaryngol Head Neck Surg. 2020;146(4):331‐337. 10.1001/jamaoto.2019.4559 32053138 PMC7042926

[20] Wilcox MB , Laranjeira SG , Eriksson TM , et al. Characterising cellular and molecular features of human peripheral nerve degeneration. Acta Neuropathol Commun. 2020;8(1):51. 10.1186/s40478-020-00921-w 32303273 PMC7164159

[21] Gomez‐Sanchez JA , Pilch KS , van der Lans M , et al. After nerve injury, lineage tracing shows that myelin and remak schwann cells elongate extensively and branch to form repair schwann cells, which shorten radically on remyelination. J Neurosci. 2017;37(37):9086‐9099. 10.1523/JNEUROSCI.1453-17.2017 28904214 PMC5597985

[22] Allodi I , Udina E , Navarro X . Specificity of peripheral nerve regeneration: interactions at the axon level. Prog Neurogibol. 2012;98(1):16‐37. 10.1016/j.pneurobio.2012.05.005 22609046

[23] Doron‐Mandel E , Fainzilber M , Terenzio M . Growth control mechanisms in neuronal regeneration. FEBS Lett. 2015;589(14):1669‐1677. 10.1016/j.febslet.2015.04.046 25937120

[24] Ydens E , Amann L , Asselbergh B , et al. Profiling peripheral nerve macrophages reveals two macrophage subsets with distinct localization, transcriptome and response to injury. Nat Neurosci. 2020;23(5):676‐689. 10.1038/s41593-020-0618-6 32284604 PMC7611025

[25] Lu SC . Regulation of glutathione synthesis. Mol Aspects Med. Feb‐apr. 2009;30(1–2):42‐59. 10.1016/j.mam.2008.05.005 PMC2704241 18601945

[26] Ursini F , Maiorino M . Lipid peroxidation and ferroptosis: The role of GSH and GPx4. Free Radic Biol Med. 2020;152:175‐185. 10.1016/j.freeradbiomed.2020.02.027 32165281

[27] Li N , Wang W , Zhou H , et al. Ferritinophagy‐mediated ferroptosis is involved in sepsis‐induced cardiac injury. Free Radic Biol Med. 2020;160:303‐318. 10.1016/j.freeradbiomed.2020.08.009 32846217

[28] Lu K , Alcivar AL , Ma J , et al. NRF2 induction supporting breast cancer cell survival is enabled by oxidative stress‐induced DPP3‐KEAP1 interaction. Cancer Res. 2017;77(11):2881‐2892. 10.1158/0008-5472.CAN-16-2204 28416489 PMC5464605

[29] Zhang DD . Mechanistic studies of the Nrf2‐Keap1 signaling pathway. Drug Metab Rev. 2006;38(4):769‐789. 10.1080/03602530600971974 17145701

[30] Sun X , Ou Z , Chen R , et al. Activation of the p62‐Keap1‐NRF2 pathway protects against ferroptosis in hepatocellular carcinoma cells. Hepatology. 2016;63(1):173‐184. 10.1002/hep.28251 26403645 PMC4688087

[31] Chen D , Tavana O , Chu B , et al. NRF2 is a major target of ARF in p53‐independent tumor suppression. Mol Cell. 2017;68(1):224‐232.e4. 10.1016/j.molcel.2017.09.009 28985506 PMC5683418

[32] Roh JL , Kim EH , Jang H , Shin D . Nrf2 inhibition reverses the resistance of cisplatin‐resistant head and neck cancer cells to artesunate‐induced ferroptosis. Redox Biol. 2017;11:254‐262. 10.1016/j.redox.2016.12.010 28012440 PMC5198738

[33] Adedoyin O , Boddu R , Traylor A , et al. Heme oxygenase‐1 mitigates ferroptosis in renal proximal tubule cells. Am J Physiol Renal Physiol. 2018;314(5):F702‐f714. 10.1152/ajprenal.00044.2017 28515173 PMC6031916

[34] Kwon MY , Park E , Lee SJ , Chung SW . Heme oxygenase‐1 accelerates erastin‐induced ferroptotic cell death. Oncotarget. 2015;6(27):24393‐24403. 10.18632/oncotarget.5162 26405158 PMC4695193

